# Implementation of a multisite, interdisciplinary remote patient monitoring program for ambulatory management of patients with COVID-19

**DOI:** 10.1038/s41746-021-00490-9

**Published:** 2021-08-13

**Authors:** Jordan D. Coffey, Laura A. Christopherson, Amy E. Glasgow, Kristina K. Pearson, Julie K. Brown, Shelby R. Gathje, Lindsey R. Sangaralingham, Eva M. Carmona Porquera, Abinash Virk, Robert Orenstein, Leigh L. Speicher, Dennis M. Bierle, Ravindra Ganesh, Debra L. Cox, R. Nicole Blegen, Tufia C. Haddad

**Affiliations:** 1 Center for Digital Health, Mayo Clinic, Rochester, MN USA; 2 Kern Center for the Science of Health Care Delivery, Mayo Clinic, Rochester, MN USA; 3 Department of Nursing, Mayo Clinic, Rochester, MN USA; 4 Department of Management Engineering and Consulting, Mayo Clinic, Rochester, MN USA; 5 Division of Pulmonary and Critical Care Medicine, Mayo Clinic, Rochester, MN USA; 6 Division of Infectious Diseases, Mayo Clinic, Rochester, MN USA; 7 Division of Infectious Diseases, Mayo Clinic, Phoenix, AZ USA; 8 Division of General Internal Medicine, Mayo Clinic, Jacksonville, FL USA; 9 Division of General Internal Medicine, Mayo Clinic, Rochester, MN USA; 10 Department of Oncology, Mayo Clinic, Rochester, MN USA

**Keywords:** Outcomes research, Rehabilitation

## Abstract

Established technology, operational infrastructure, and nursing resources were leveraged to develop a remote patient monitoring (RPM) program for ambulatory management of patients with COVID-19. The program included two care-delivery models with different monitoring capabilities supporting variable levels of patient risk for severe illness. The primary objective of this study was to determine the feasibility and safety of a multisite RPM program for management of acute COVID-19 illness. We report an evaluation of 7074 patients served by the program across 41 US states. Among all patients, the RPM technology engagement rate was 78.9%. Rates of emergency department visit and hospitalization within 30 days of enrollment were 11.4% and 9.4%, respectively, and the 30-day mortality rate was 0.4%. A multisite RPM program for management of acute COVID-19 illness is feasible, safe, and associated with a low mortality rate. Further research and expansion of RPM programs for ambulatory management of other acute illnesses are warranted.

## Introduction

At the onset of the COVID-19 pandemic and executive shelter-in-place orders in March 2020, there was an urgent need to develop new ways to support ambulatory patients with SARS-CoV-2 infection at risk for severe COVID-19 illness, decompress hospitals and emergency departments (ED), and preserve personal protective equipment (PPE)^1^. In response, global health care systems were forced to either create new telehealth and virtual care-delivery models or drive the adoption of existing and repurposed products and services^2^. In the United States, telehealth and virtual care expanded rapidly, which was enabled by changes to regulatory, licensure, and reimbursement requirements by both the federal government and private payers in response to the public health emergency^3–5^.

Mayo Clinic leveraged and rapidly scaled mature telehealth and virtual care services in response to the pandemic in support of patients with serious and complex conditions^6,7^. Additionally, new services with existing products were developed to support the clinical management of patients with acute COVID-19^7^. One such example was the utilization of the existing remote patient monitoring (RPM) technology with its operational infrastructure and clinical resources.

**Fig. 1:**
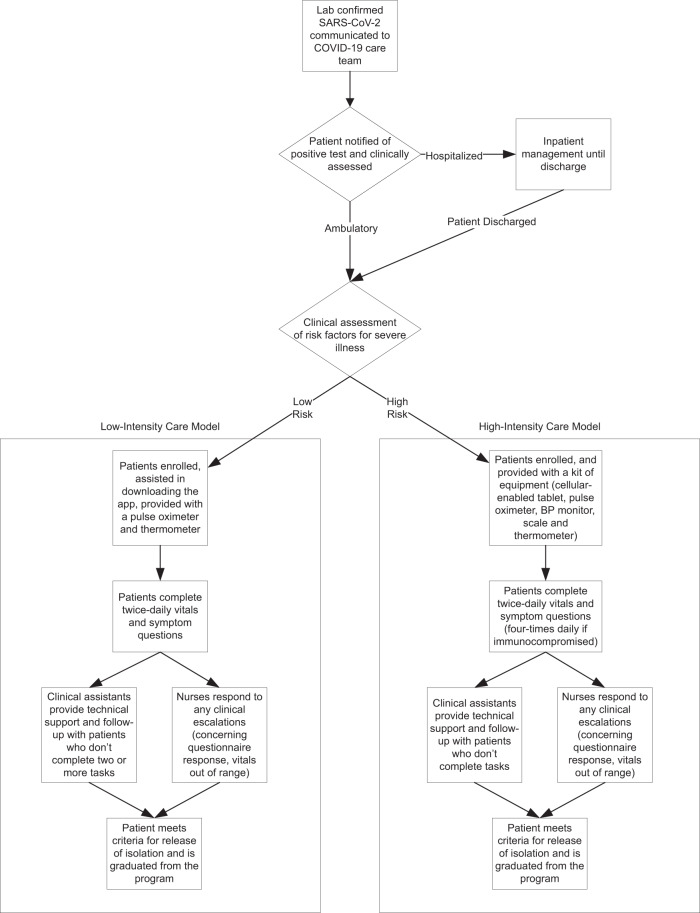
Clinical workflow for patient identification and eligibility for the COVID-19 RPM care models. Patients at high risk for severe COVID-19 illness were eligible for the high-intensity care model if they had one or more of the following: age >65 years, diabetes, current smoker, BMI >40, chronic liver disease, chronic lung disease, congestive heart failure, active cancer therapy, bone marrow or solid organ transplant, other immunocompromised state, end-stage renal disease. Additionally, patients were eligible if they were hospitalized for COVID-19 without one of these risk factors, but experienced one of the following: hospital length of stay ≥7 days, ICU admission, cardiac complications, need for mechanical ventilation or dialysis, need for oxygen supplementation at discharge, and receipt of remdesivir upon discharge.

Initially developed to support patients with complex, chronic conditions, such as congestive heart failure and chronic obstructive pulmonary disease (COPD), the RPM framework was rapidly adapted to support patients with COVID-19—a novel, acute, self-limited disease with variable clinical presentations and complications, as well as the unique requirement for self-isolation during the illness and recovery phase. The COVID-19 RPM program was developed by institutional leaders from the Mayo Clinic Center for Digital Health in partnership with a multidisciplinary team of COVID-19 physician experts. Clinical workflows were designed (Fig. 1), and the RPM operations team facilitated multisite program implementation with hospital and ambulatory COVID-19 care teams from the three main campuses in the Midwest, Southwest (Scottsdale, Arizona), and Southeast (Jacksonville, Florida) regions of the United States^8^. The Midwest region included the Rochester, Minnesota campus, as well as the community-based, affiliated Mayo Clinic Health System (MCHS) sites spanning Southern Minnesota, Western Wisconsin, and Northern Iowa. Thus, the patients eligible for RPM program participation were from geographically diverse regions in the United States, including rural and urban settings, and served by tertiary and quaternary referral centers, as well as community-based primary care practices.

Two separate COVID-19 RPM care models with differing technology capabilities and monitoring intensities were utilized to support patients at low- and high-risk for severe illness (Table 1). The technology-enabled monitoring comprised of twice-daily patient-reported symptom assessments and vital signs obtained from peripheral medical devices, including pulse oximeters, thermometers, and blood-pressure monitors. Centralized RPM-registered nurses (RNs) responded to the technology-generated alerts and utilized standardized care pathways for clinical assessments and patient management, including escalation to the COVID-19 care team physicians. Other connected health solutions were utilized to support patients with suspicion for COVID-19, and the established RPM program for patients with complex, chronic conditions was maintained.

**Table 1:** COVID-19 RPM program care model differences.

Characteristic	Low-Intensity Care Model	High-Intensity Care Model
Patient profile	• No risk factors for severe COVID-19 illness • Low symptom burden	• One or more risk factors for severe COVID-19 illness • Moderate-to-high symptom burden
Components	Care plan delivered through patient owned smart phone or tablet via the Mayo Clinic app. • Tasks assigned via notifications. Manual entry of self-reported physiologic data, questionnaires, and symptom assessments. Embedded decision trees and logic to generate alerts.	Care plan delivered through the Mayo Clinic supplied, cellular-enabled tablet. •Bluetooth-enabled medical devices passively collect physiologic data. Manual entry of self-reported questionnaires and symptom assessments.
Mayo-supplied equipment	• Commercial-grade thermometer and pulse oximeter • Optional (patient-owned): blood pressure monitor	• Cellular-enabled tablet • Clinical grade, Bluetooth-enabled blood pressure monitor, weight scale, pulse oximeter, and thermometer • Optional: peak flow meter, glucometer
Vital signs	Patient-reported (manual entry), twice per day • Temperature: >100.9°F • Heart Rate: >105 bpm • Oxygen saturation: <90% • Systolic Blood Pressure: <100 mmHg • Diastolic Blood Pressure: None • Respiratory Rate: >20 breaths per minute	Patient-reported (passively collected), twice per day; can be individualized. • Temperature: <90 °F or >100.9 °F • Heart Rate: <50 or >115 BPM • Oxygen saturation: <94% •Systolic Blood Pressure: <90 or >180 mmHg • Diastolic Blood Pressure: <50 or >110 mmHg
Care team engagement	• Welcome call: Introduction to RPM equipment/program, contact numbers, when to seek emergency care if needed • Symptom calls: In response to abnormal vital signs or new/worsening symptoms registered within the Mayo Clinic mobile app	• Welcome call: Introduction to RPM equipment/program, initial symptom assessment, contact numbers, when to seek emergency care if needed • Symptom calls: In response to abnormal vital signs or new/worsening symptoms registered on the tablet • Graduation (Day 10 or 20 from symptom onset): Symptom assessment for improvement, release from isolation and return to the community/work
Monitoring	• InBasket message (i.e., EHR-integrated message) alerts to centralized RPM RN team based on predetermined care plan logic; RN escalates to Mayo-led centralized COVID-19 care team as needed	• Prioritized alerts, based on patient-specific alert parameters, presented to centralized RPM RN team through a dedicated web-based patient management console; RN escalates to Mayo-led centralized COVID-19 team as needed
Capacity	1:50 (nurse to patient ratio)	1:30 (nurse to patient ratio)
Reimbursement	Non-billable	Billable service under code 99453, 99454, 99457, and 99458

While the value proposition for RPM in the management of chronic conditions is to reduce acute care utilization and hospital admissions,^9^ during the COVID-19 RPM program development, it was recognized that emergency department (ED) visits and hospitalizations would be unavoidable when monitoring patients at risk for severe illness; however, it was hypothesized that early detection of adverse health trends and early supportive care interventions in patients with acute illness could favorably alter their disease trajectory.

Prior research has demonstrated that an RPM program for patients with symptoms concerning for COVID-19 was safe and effective; however, 92% of patients did not test for COVID-19, only 1% tested positive, and clinical outcomes were not reported^10^. In a separate study, an RPM program for patients discharging from the hospital after acute COVID-19 illness was associated with fewer ED visits and readmissions^11^; however, the corresponding clinical outcomes were not reported. In addition to these limitations, at the time of this report, there are no studies published evaluating an RPM program for ambulatory management of COVID-19 from diagnosis through the acute phase and recovery. As such, it remains unknown if patients will engage with the technology and virtual care team while acutely ill, and how the home-based care model may impact patient safety and clinical outcomes. The majority of published COVID-19 RPM studies have also been limited to a cohort of patients from a single site or region and most commonly within urban populations; thus, the feasibility of a multisite/multiregion model with centralized virtual care team management encompassing both urban and rural locations remains unknown.

Herein we report the Mayo Clinic experience with the development and implementation of a large-scale, multisite, interdisciplinary COVID-19 RPM program, as well as the results of a study to evaluate the impact of this care delivery model^10,12^. This study aims to address the noted gaps in the medical literature by evaluating how an RPM program can be effectively adapted at scale to facilitate multisite and multi-regional, ambulatory management of a large population of patients with an acute condition. Our study objective was to determine the feasibility and safety of the COVID-19 RPM program as measured by patient engagement with the technology, rate of alerts and escalations managed by virtual care teams, acute care resource utilization rates, and patient clinical outcomes.

## Results

### Patient cohort description

**Fig. 2:**
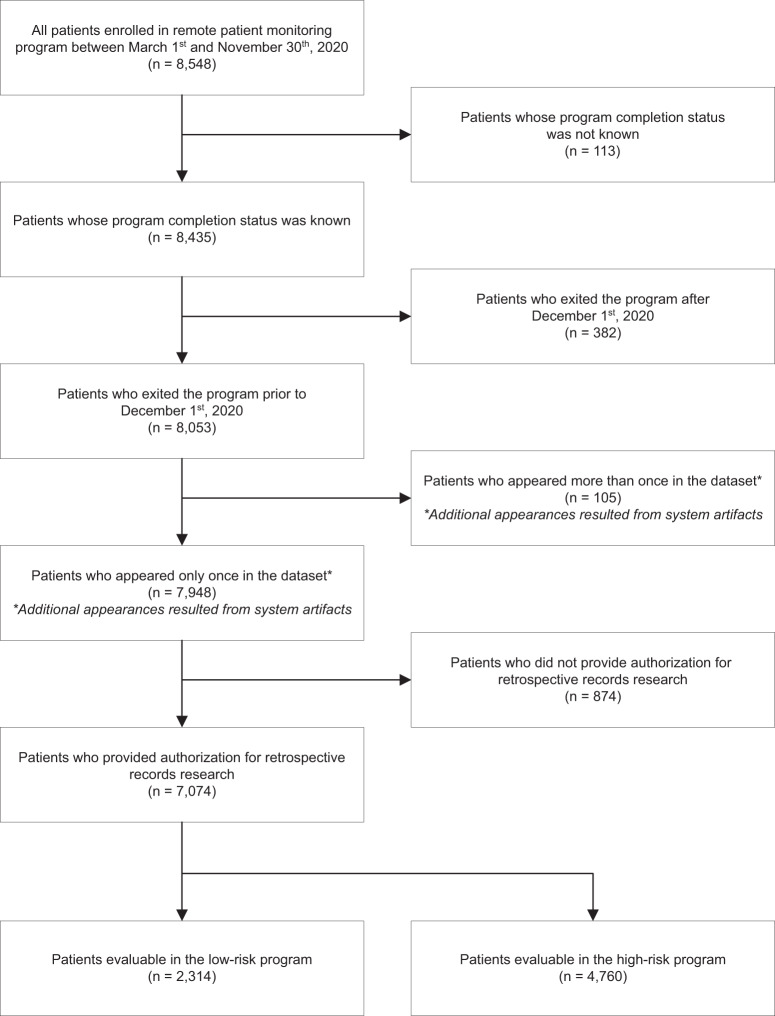
Patient flow diagram for the RPM program. Patient enrollment, disposition, and authorization for retrospective record research for the final analysis.

Over eight months from March 26^th^ to November 30^th^, 2020, the COVID-19 RPM program enrolled 8548 patients with COVID-19 in the ambulatory setting, including 2728 (31.9%) in the low-intensity care model and 5820 (68.1%) patients in the high-intensity care model. Of the enrolled patients, 1474 were not included in the final analysis for reasons described in Fig. 2 flow diagram, including 874 who did not provide authorization for retrospective record research.

**Fig. 3:**
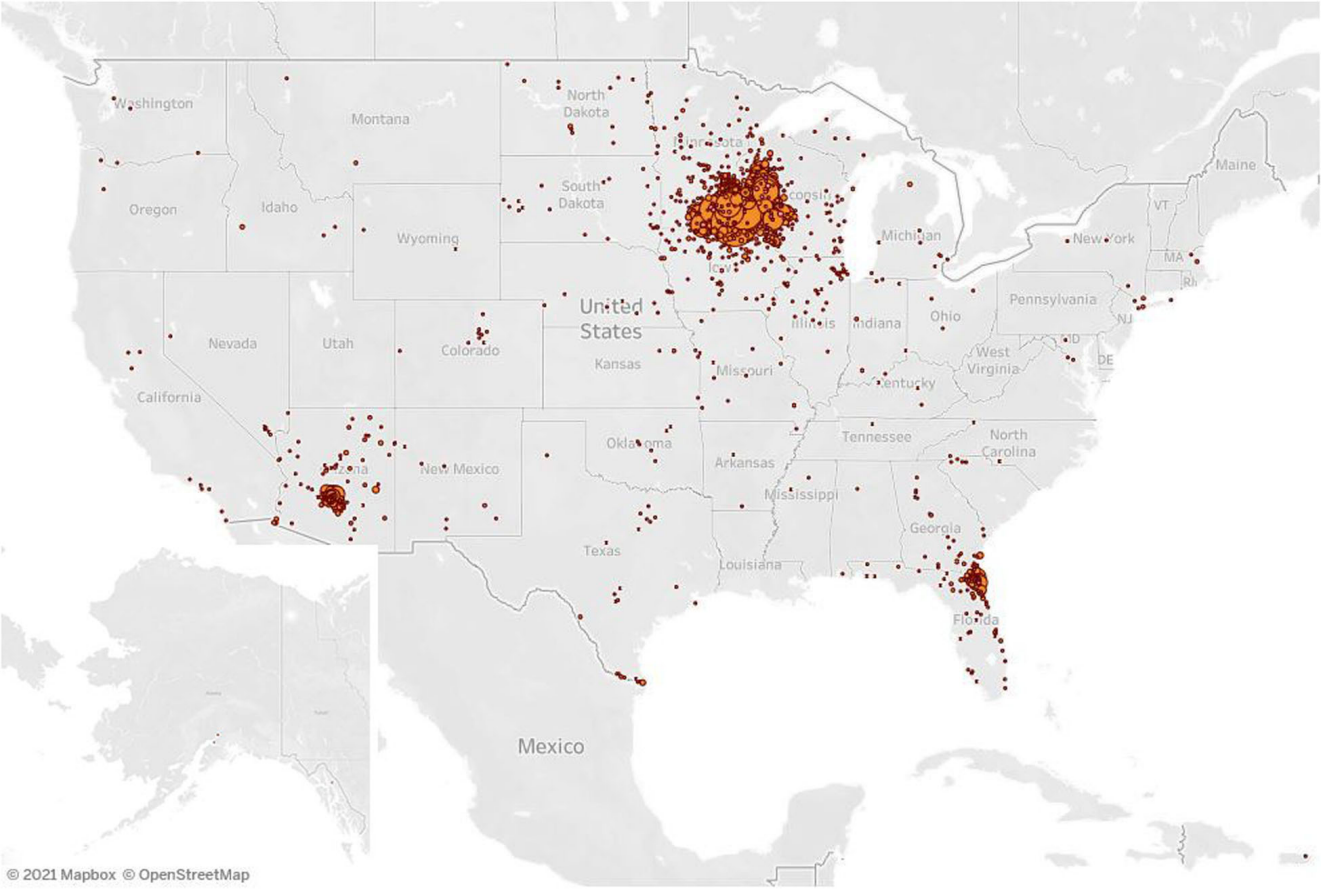
Geographic distribution of patients managed with the high-intensity RPM care model. Patients participating in the high-intensity RPM program were from 39 states across the United States. When including the low-intensity care model, coverage was extended to an additional two states (Louisiana and Utah). The RPM program participants were centrally monitored by Midwest RPM RNs and regionally managed by Midwest, Southwest, and Southeast COVID-19 care team physicians and advanced practice providers. © Mapbox, © OpenStreetMap. Prints use map data from Mapbox and OpenStreetMap and their data sources. To learn more, visit https://www.mapbox.com/about/maps/ and http://www.openstreetmap.org/copyright.

The cohort for the final analysis (*n* = 7074) included 2314 patients enrolled to the low-intensity monitoring and 4760 patients to the high-intensity monitoring care models. Table 2 summarizes detailed patient demographics and characteristics. In the overall cohort, the median patient age was 28 years for low-intensity monitoring and 58 years for high-intensity monitoring. There were slightly more women than men in both care models. The overall population was diverse with racial/ethnic minority patients comprising 27.5% (*n* = 1866) of all patients in which race/ethnicity data were available (*n* = 6785). Within the high-intensity monitoring, English was not the primary language for 15.5% of patients, and of those, a breakdown by primary language included 70.0% Spanish, 12.8% Somali, and 17.2% other. Of those in the low-intensity monitoring, English was not the primary language for 5.3% of patients, 73.8% of which spoke a language other than Spanish or Somali. Differences in language distribution between programs was expected as the technology for the high-intensity monitoring provided content in languages other than English, including Spanish. While patients were diagnosed at one of the three main Mayo Clinic campuses or four MCHS regions in the Midwest, their residential addresses spanned 41 of 50 United States (Fig. 3). Of those in the high-intensity monitoring model, 54.2% were enrolled from the community-based MCHS sites, and 44.9% had a Mayo Clinic primary care provider (Table 2).

**Table 2:** Patient demographics and characteristics.

Characteristic	Low-intensity enrollee (*n* = 2314)	High-intensity enrollee (*n* = 4760)
*Age, years*		
Mean (SD)	32.9 (13.8)	55.7 (18.3)
Median	28	58
Range	18–84	17–101
*Age distribution*		
18–34	1487 (64.3%)	766 (16.1%)
35–49	450 (19.4%)	949 (19.9%)
50–64	330 (14.3%)	1303 (27.4%)
65–74	39 (1.7%)	965 (20.3%)
74+	8 (0.3%)	777 (16.3%)
*Sex*		
Female	1280 (55.3%)	2461 (51.7%)
Male	1030 (44.5%)	2298 (48.3%)
Missing	4 (0.2%)	1 (0.0%)
*Married*	749 (32.4%)	2855 (60.0%)
*Race/Ethnicity*		
Hispanic (all races)	225 (9.7%)	720 (15.1%)
White, Non-Hispanic (NH)	1566 (67.7%)	3353 (70.4%)
Black, NH	187 (8.1%)	284 (6.0%)
Asian, NH	75 (3.2%)	132 (2.8%)
All other, NH	90 (3.9%)	153 (3.2%)
Missing	171 (7.4%)	118 (2.5%)
*Primary language*		
English	2192 (94.7%)	4023 (84.5%)
Spanish	12 (0.5%)	516 (10.8%)
Somali	20 (0.9%)	94 (2.0%)
Other	90 (3.9%)	127 (2.7%)
*Paneled to Mayo Clinic primary care*		
No	1177 (50.9%)	2138 (44.9%)
Yes	1137 (49.1%)	2622 (55.1%)
*RPM enrollment by Mayo Clinic Region*^a^		
MCHS, Northwest Wisconsin	–	677 (14.2%)
MCHS, Southeast Minnesota	–	913 (19.2%)
MCHS, Southwest Minnesota	–	564 (11.8%)
MCHS, Southwest Wisconsin	–	426 (8.9%)
Rochester	–	1079 (22.7%)
Arizona	2 (0.1%)	659 (13.8%)
Florida	15 (0.6%)	439 (9.2%)

^a^Data for enrollment by region of 2297 Midwest patients in the low-intensity monitoring care model is not available as all orders for the program originated from the centralized Midwest care team providers in Rochester.

Monthly enrollment to both care models over the eight-month study period was uniformly distributed with a few notable exceptions. There was limited enrollment in April as the program was sequentially implemented across the three regions and the overall COVID-19 case volumes were low in the Midwest. Higher monthly enrollment was present during the Summer (June–July) Southern surge and the Fall (October–November) Midwest surge in the United States. The low-intensity monitoring care model was discontinued in September 2020 following program analysis, and details related to this are provided in the “Discussion”.

In the overall cohort, most patients received their COVID-19 diagnosis in the outpatient setting; however, 26.7% of those in the high-intensity monitoring were diagnosed in the hospital and enrolled upon discharge. Patient comorbidity information is summarized in Table 3. The patients enrolled in the high-intensity monitoring care model had more COVID-19 risk factors for severe illness and baseline comorbidities reflective of program-eligibility criteria. Of the 35.3% of patients in this cohort without a risk factor, reasons for inclusion in the high-intensity monitoring were related to the technology platform providing Spanish language capability, flexible parameter alerts adjusted for pregnant patients, and program access for patients without a mobile device (required for low-intensity monitoring).

**Table 3:** COVID-19 related characteristics and risk factors for severe illness.

Characteristic	Low-intensity enrollee (*n* = 2314)	High-intensity enrollee (*n* = 4760)
*Ordering test location*		
Inpatient	89 (3.8%)	1271 (26.7%)
Outpatient	2215 (95.7%)	3289 (69.1%)
Missing	10 (0.4%)	200 (4.2%)
*Month of COVID test result*		
March	1 (0.0%)	25 (0.5%)
April	76 (3.3%)	124 (2.6%)
May	343 (14.8%)	338 (7.1%)
June	577 (24.9%)	662 (13.9%)
July	531 (22.9%)	692 (14.5%)
August	499 (21.6%)	443 (9.3%)
September	277 (12.0%)	521 (10.9%)
October	0 (0.0%)	964 (20.3%)
November	0 (0.0%)	785 (16.5%)
*COVID-19 risk factors per patient (sum)*		
Mean (SD)	0.0 (1.0)	1.0 (4.0)
Median	1	2
*Elixhauser score*		
Mean (SD)	0.5 (1.0)	3.2 (3.2)
Median	0	2
*Risk factors for severe COVID-19 illness*		
Age >65	43 (1.9%)	1618 (34.0%)
Diabetes	34 (1.5%)	1005 (21.1%)
COPD/Emphysema	4 (0.2%)	317 (6.7%)
Asthma	61 (2.6%)	645 (13.6%)
Chronic lung disease	79 (3.4%)	747 (15.7%)
Congestive heart failure	4 (0.2%)	395 (8.3%)
Coronary artery disease	8 (0.3%)	478 (10.0%)
Current smoker	99 (4.3%)	797 (16.7%)
Active chemotherapy	1 (0.0%)	119 (2.5%)
Bone marrow transplant	0 (0.0%)	29 (0.6%)
Solid organ transplant	0 (0.0%)	71 (1.5%)
Immunocompromised	3 (0.1%)	261 (5.5%)
End stage renal disease	8 (0.3%)	625 (13.1%)
End stage liver disease	7 (0.3%)	235 (4.9%)
Obesity	146 (6.3%)	941 (19.8%)
None of the above	1987 (85.9%)	1680 (35.3%)

### Patient engagement and RPM monitoring metrics

Definitions for endpoints associated with the RPM program monitoring and digital exchanges are provided in Table 4. Patients enrolled in the COVID-19 RPM program were actively engaged with the monitoring technology as summarized by the compliance rates, alerts, and care-escalation data in Table 5.

**Table 4:** Definitions of endpoints for COVID-19 RPM program engagement and digital exchanges.

Measurement	Description
Engagement (monitoring rate)	The number of patients who engaged and received at least one day of monitoring was tabulated. The engagement calculation = total patients engaged/total number of patients enrolled.
Program duration	The number of days (mean and median) monitored across the total engaged patient population. The program duration calculation = total number of days from enrollment to the date of having either completed or dropped out of the RPM program.
Compliance (completed task rate)	When patients do not complete assigned tasks for self-reporting of vital signs or symptoms, a missed task notice is generated. Total scheduled tasks, as well as total tasks missed and completed were tabulated. The compliance calculation = total tasks completed/total scheduled tasks.
Alert rate	When patient-reported vital signs and symptoms are out of a prespecified normal range, an alert is generated. The total number of alerts was tabulated. The alert rate calculation = total alerts/total scheduled tasks.
Escalation rate	For each alert, an RPM RN completes a clinical assessment to determine if further escalation to a care team provider or referral to the ED are indicated. The total number of escalations were tabulated. The escalation rate calculation = total number of escalations/total alerts.
Program completion rate	The number of patients who completed their assigned program (i.e., reached the point of clearance/graduation) was tabulated. The program completion rate = total number of patients graduated/total patients enrolled.

**Table 5:** COVID-19 RPM program digital engagement, compliance, alerts, and escalations.

Results	Low-intensity enrollee (*n* = 2314)	High-intensity enrollee (*n* = 4760)
Engagement	80.0%	78.4%
Program duration (days)	14.6 (median 9)	13.2 (median 12)
Compliance	61.6%	72.5%
Missed tasks per patient	3.87	4.45
Alert rate	3.2%	19.6%
Alerts per patient	1.87	3.17
Escalation rate	9.4%	18.8%
Escalations per patient	1.46	3.04
Program completion rate	N/A	72.1%

#### Low-intensity monitoring

Of all patients enrolled, 80.0% had at least one day of engagement with the interactive care plan. Median program duration was 9.0 days. Compliance with care plan tasks was 61.6%. Out of all tasks, 3.2% generated an alert for out-of-range symptoms or vital sign parameters. The RPM RNs conducted a clinical assessment for each alert received, and of these alert assessments, 9.4% required escalation to a COVID-19 care team provider.

#### High-intensity monitoring

Of all patients enrolled, 78.4% had at least one day of engagement with the RPM equipment. Median program duration was 12.0 days. Of those who were actively monitored, 72.1% completed the program through graduation. Compliance with care plan tasks was 72.5%. Out of all tasks, 19.6% generated an alert for out-of-range symptoms or vital sign parameters. After RPM RN clinical assessment for each alert, 18.8% required escalation of care to a COVID-19 care team provider.

### Acute care resource utilization

For patients enrolled to the RPM program following hospital discharge, the originating hospitalization and any associated ED visit or intensive care unit (ICU) admission were excluded from the acute care resource utilization reported in this analysis. Of all patients, 11.4% (*n* = 809) experienced an ED visit, and 9.4% (*n* = 663) were admitted to a hospital within 30 days of enrollment (among patients diagnosed in the outpatient setting) or within 30 days of discharge (among patients diagnosed in the hospital setting). Additionally, there were 78 ICU stays among the cohort. The mean hospital length of stay was 3.0 days (SD 5.1) and 6.4 days (SD 7.6) for the low- and high-intensity monitored patients, respectively. Most acute care utilization was incurred by patients participating in the high-intensity RPM care model as outlined in Table 6. They accounted for 82.6% of ED visits, 95.0% of hospital admissions, and 93.6% of ICU admissions.

**Table 6:** Acute care resource utilization and clinical outcomes.

Acute care resource utilization	Low-intensity enrollee (*n* = 2314)	High-intensity enrollee (*n* = 4760)
*Emergency department (ED) visits*		
N	141	668
Unique patients (≥1 ED visit)	114 (4.9%)	550 (11.6%)
Unique patients (>1 ED visit)	22 (1.0%)	96 (2.0%)
*Hospital admissions*		
N	33	630
Unique patients (≥1 admission)	29 (1.3%)	543 (11.4%)
Unique patients (>1 admission)	4 (0.2%)	77 (1.6%)
*Average length of stay (days, for those admitted)*		
Mean (SD)	3.0 (5.1)	6.4 (7.6)
Median	1	5
*Intensive care unit (ICU) admissions*		
N (%)	5 (0.2%)	73 (1.5%)
Total days (for those with ICU admission)		
Mean (SD)	3.0 (3.5)	6.8 (9.4)
Median	1	4
*Clinical outcomes*		
Mortality	0 (0.0%)	27 (0.6%)
Complications		
Acute kidney injury	0 (0.0%)	227 (4.8%)
Venous Thromboembolism	3 (0.1%)	154 (3.2%)
Cardiogenic shock or myocardial infarction	1 (0.0%)	44 (0.9%)

All data are reported for events that occurred within 30 days of RPM program enrollment for outpatient diagnosis or 30 days from discharge of a hospitalized patient.

### Clinical outcomes

The 30-day rate of COVID-19 complications was less than 1% in those receiving low-intensity monitoring. Of those in the high-intensity care model, the most common complications experienced were a venous thromboembolic event in 3.2% of patients and acute kidney injury in 4.8%. There were 27 deaths among all patients (0.4% mortality rate), all of whom were monitored in the high-intensity care model. Table 6 provides a summary of these data with reporting for both care models.

## Discussion

Leveraging established low- and high-intensity technology products, operational infrastructure, and centralized nursing coordinators, the COVID-19 RPM program was rapidly designed and implemented across all three Mayo Clinic campuses and community-based regional MCHS sites. Over the eight months following the COVID-19 pandemic emergency declaration, 8548 patients enrolled in the RPM program and 7074 were evaluable for this feasibility and safety analysis. The size of the patient cohort in this analysis is substantially larger than prior reports of RPM for management of patients with COVID-19 and among the first of its size for RPM in the management of an acute condition. It is also responsive to the request for more studies of large-scale RPM implementations, a recommendation made after a meta-analysis of RPM studies revealed only 23.5% of the 272 published studies involving an *n* greater than 50^13^.

There are several strengths and unique features associated with this COVID-19 RPM program: (1) high- and low-intensity monitoring care models, (2) centralized RPM RN care coordinators to provide 24/7 monitoring and digital frontline care, (3) regional COVID-19 care team physician and advanced practice provider oversight, (4) standardized care pathways to optimize management aligned with evolving CDC guidance and Mayo Clinic expert opinion, (5) expansive reach to care for patients across 41 US states, and (6) a diverse patient population with 27.5% racial/ethnic minority representation. Being an established Mayo Clinic patient was not a requirement for COVID-19 RPM program eligibility. Because of this, access to care was expanded, and the patient diversity in the RPM cohort was more reflective of the regional catchment areas than the overall Mayo Clinic patient population.

Initial clinical assessments in response to alerts were managed by the RPM RNs who validated abnormal physiologic data, evaluated concerning symptoms, and provided supportive care recommendations when appropriate. With this nurse-led care model, only 9–18% of alerts required escalation to a small core team of physicians. The standardized care pathways also allowed for rapid, scalable program adjustments to accommodate changes in patient eligibility, alert parameters, as well as advances in COVID-19 treatment. For example, outpatient administration of remdesivir and antispike monoclonal antibody administration was implemented as part of the RPM program on November 10^th^ and 13^th^, respectively, only days to weeks after they became clinically available.

An additional strength of this RPM program was the intentional efforts to incorporate interventions that supported patient access and engagement and mitigated digital health disparities.^14^ A few examples included development of protocols to assess patients’ technical readiness following enrollment, including written and oral instructions in their native languages (to include Spanish, Arabic, Somali, and Hmong); RPM equipment delivery directly to patients’ home or recovery location; and, utilization of a technology platform with integrated cellular-enabled connectivity to support those without Internet access (high-intensity model). Support staff also monitored patient engagement and utilized both asynchronous and synchronous communication (e.g., secure messages and telephone calls) to check in with patients and offer nonclinical assistance. These interventions contributed to an overall patient engagement rate of 78.9%. There is scant literature on patient engagement rates with RPM technology for large, diverse patient cohorts^12^, and this study advances the study of RPM by demonstrating that patients will engage with the technology and a remote centralized care team, even while acutely ill. It further establishes an engagement rate from which others could benchmark in subsequent studies.

Patients in the low-intensity RPM care model were generally young adults with an ambulatory diagnosis and limited comorbid conditions or risk factors for severe illness. Of those enrolled, 80.0% engaged with the care plan through the Mayo mobile app. Only 3.2% of tasks to self-report symptoms and vitals led to alerts, of which only 9.4% were escalated to a care team physician after the initial RPM RN assessment. Acute care resource utilization was low, and none of the patients died. At the time these data were initially analyzed in September 2020, the RPM operations and clinical leaders, along with the three regional COVID-19 care team physician leaders, made the decision to discontinue the low-intensity RPM care model, given the low alert rate and overall favorable prognosis and clinical outcomes observed in this patient cohort.

As designed, the high-intensity RPM care model supported an older patient population, the majority of whom had one or more comorbid conditions and/or risk factors for severe COVID-19 illness. Of those enrolled, 78.4% engaged with the tablet and peripheral medical devices. Compliance with assigned tasks was higher in this group (72.5%) relative to the low-intensity monitoring cohort, which may be due to higher patient acuity and complexity and/or more user-friendly, preconfigured RPM technology with Bluetooth-enabled devices to passively collect vital sign data. The 19.6% alert rate was higher in this cohort, reflective of the increased symptom burden and vital sign instability associated with COVID-19 in patients at risk for severe illness, as well as the lower threshold to alert for declining oxygen saturation in this cohort. The percentage of alerts that required escalation to a care team physician following RPM RN assessment was twice as high in the high-intensity RPM model, and these patients experienced the majority of all ED visits, hospitalizations, and ICU admissions. Despite this, the mortality rate in this high-risk patient population was extremely low at 0.6%, below rates reported by the CDC as well as in large observational studies and clinical trials^15,16^.

The results of this study suggest that RPM for management of an acute condition, COVID-19, is feasible, safe, and associated with excellent clinical outcomes. Notably this was observed among patients at risk for severe illness. Such findings provide early evidence in support of 2021 changes made to the Centers for Medicare & Medicaid Services physician fee schedule final rule to expand reimbursement for RPM services to patients with acute conditions^17^. They could additionally be leveraged to persist some of medical licensure and regulatory changes that enabled telehealth and virtual care to be provided across state lines in response to the public health emergency^18,19^. These were major barriers to telehealth and virtual care adoption prior to the COVID-19 pandemic, and these study results may help inform other needed healthcare policy changes to sustain adoption beyond it.

Among the limitations to the program and study is that they represent the experience of a single institution. That said, the Mayo Clinic enterprise includes both tertiary and quaternary referral centers, as well as community-based practices, coverage across three U.S. geographic regions, and a combination of rural and urban-based locations. Additional studies will be required to demonstrate the reproducibility of the program implementation at other clinical sites and health care systems, and the authors have provided important program development and implementation details in the methodology with the aim to make the program framework transparent and accessible to others for this purpose. A key criticism of the technology platforms for both monitoring care models did not offer language support beyond English and Spanish. To address this, medical interpreters were utilized for technical questions and to support interactions with RPM nursing and COVID-19 care team providers. In order to facilitate equitable access and engagement with RPM solutions in the future, we must demand that the technology vendors enable more diverse language offerings for their products.

Given that this is a retrospective cohort study, it remains unknown how COVID-19 patient management with the RPM program and care-delivery models compares with “usual care” in terms of clinical outcomes, patient experience, acute care resource utilization, and cost of care. A matched case-control study to evaluate the program is planned. These findings and patient satisfaction survey results will be reported in a future manuscript. Prospective, pragmatic trials will ultimately provide the strongest evidence in support for RPM adoption and expansion, as well as the needed health policy, regulatory, and reimbursement changes to enable its sustainability.

## Methods

### Setting

Mayo Clinic is a nonprofit, specialty group practice, with integrated research, education, and clinical practice activities. Over 1.2 million patients are served annually across three main campuses (Rochester, Minnesota; Scottsdale, Arizona; and Jacksonville, Florida) and over 70 Midwest community-based hospitals and clinics in Southern Minnesota (SE MN and SW MN regions), Northern Iowa (included in the SE MN region), and Western Wisconsin (NW WI and SW WI regions) that comprise the MCHS^20,21^.

The Center for Connected Care was established in 2012 (now integrated within the Center for Digital Health) as a vital practice strategy to enable Mayo Clinic physicians and care teams to deliver health care virtually so that referring providers and patients have access to Mayo Clinic’s expertise and knowledge through various technologies and integrated practice models at the right time and in the right place. Telehealth and virtual care services supported by Connected Care include the following four product lines: asynchronous (including patient online services, secure messaging, referring provider portals, and virtual consults), synchronous video telemedicine (including outpatient and inpatient video telemedicine, as well as advanced acute telemedicine services), RPM (including interactive care plans), and mobile platform^7^. RPM was established in 2015 to be an “Area of Excellence,” ensuring standardized and scalable RPM services with centralized program management, clear governance and oversight, standardized clinical practice and reporting, and partner and vendor management.

### RPM solution overview

RPM uses digital applications and devices to collect, analyze, and record physiologic and other patient-generated health data outside of a clinical setting^12,13^. Use of this technology allows for early detection of trends that could result in adverse health events and creates the opportunity to provide individualized feedback to patients and healthcare teams^12^. The Mayo Clinic RPM service line’s overall strategy was to leverage technology that engages patients as active participants in their care with the goals of improving patient experience, fostering self-management, reducing healthcare resource utilization, reducing cost of care, and ultimately to impact clinical outcomes positively. Included in this strategy was a shared responsibility for clinical program development with the Mayo Clinic clinical practice and development of scalable, standardized products and solutions by the RPM staff.

The RPM service line offers two levels of monitoring intensity, each with unique value propositions, technology solutions, and models of care delivery. All conditions supported by the RPM program, through either care-delivery model, are anchored by care plan tasks that deliver educational content and prompt symptom reporting and physiologic data entry. These RPM care plans are developed by multidisciplinary teams that comprise of clinical subject matter experts from the practice (e.g., physicians, nurses, pharmacists, and therapists) and RPM program staff (e.g., physicians, nurses, business analysts, health system engineers, content editors, user experience designers, and information technology liaisons). All care plans are furthermore endorsed across all Mayo sites to ensure adoption of best practices and promote standardization of care.

### Technology platforms and clinical care models

The high-intensity RPM care model focuses on patients whose complex care needs require daily monitoring with clinical grade, in-home equipment and centralized RPM RN support. It is designed to support patients with unstable medical conditions or those at risk for serious complications of their treatment. Once enrolled, the patient is sent a technology package comprising of a cellular-enabled tablet preloaded with a vended clinical RPM software (Resideo Life Care Solutions) and preconnected, Bluetooth-enabled devices (blood-pressure cuff and monitor, pulse oximeter, thermometer, and scale) to passively collect physiologic data. The tablet notifies patients to perform vital sign measurements and complete condition-specific symptom assessments at appropriate intervals, at least once daily. Responses trigger alerts based on predetermined parameters, and all data are integrated with the electronic health record (EHR), Epic. Key to this RPM model is a centralized team of RPM RNs who provide daily monitoring and education, respond to alerts and missed tasks, leverage standardized decision trees and protocols for interventions, and escalate to the appropriate managing clinician or department as necessary. The high-intensity RPM care model was initially developed as a 90-day program for chronic condition management (congestive heart failure, chronic obstructive pulmonary disorder, hypertension, coronary artery disease, and diabetes) or a 30-day program for select post-surgical/procedural support. All patients must be paneled in either primary or specialty care and at risk for acute care resource utilization, prolonged hospital stay, or hospital readmission. This model was implemented at all Mayo Clinic hospitals and ambulatory primary care practices. Prior to the COVID-19 pandemic, the daily census was 350 patients and over 3000 patients had been served since its inception in 2016 and phased rollout through 2019.

The low-intensity RPM care model (or interactive care plan) is delivered through the Mayo Clinic mobile app on the patient’s smartphone or tablet, and care oversight is decentralized to patients’ primary or specialty care team. It is designed to support clinically stable patients with a new diagnosis, limited medical event, or ongoing post treatment rehabilitation and surveillance. Care plans delivered through this model are designed within existing electronic health record (EHR) functionality and integrated with the Mayo Clinic mobile app. Once enrolled in a care plan, patients receive notifications when tasks are due. Completed education content viewing, symptom assessments, questionnaires, and physiologic data submitted by the patients are available in the EHR and through care team dashboards. Decision trees and logic are embedded within each care plan. If patient-generated health data are outside of predetermined parameters, patients will receive education to facilitate self-management, or for more serious deviations, alert messages will be directed to the managing care teams. Prior to the COVID-19 pandemic, eight interactive care plans were developed, one of which was implemented in the cardiology practice to support systolic heart failure patients. Other care plan implementations were delayed due to the diversion of resources to support the pandemic response.

### COVID-19 RPM program

Mayo Clinic leveraged the RPM program and both high- and low-intensity RPM care models to manage patients with COVID-19 in the ambulatory setting (see Table 1 for program comparison). The high-intensity model aimed to support patients with one or more risk factors for severe COVID-19 illness as defined by the CDC and expert consensus. In contrast, the low-intensity model was adapted to support all patients, regardless of risk and to provide redundancy if there were supply chain issues with the vended technology package.

To meet the unique needs of patients with acute COVID-19, a multidisciplinary team comprising of RPM clinical nurse specialists, physicians, patient education specialists, and COVID-19 physician experts from the Divisions of General Internal Medicine (GIM), Infectious Disease, and Pulmonary Medicine was rapidly formed. Over a ten-day sprint, the team created patient symptom assessments and established vital signs for patient monitoring and parameters for alerts (see Table 1), as well as evidence-based, best-practice decision trees, order sets, and educational content to serve as the foundation for both the high- and low-intensity COVID-19 RPM care models.

Features common across both care models include the use of peripheral medical devices (pulse oximeters and thermometers) for vital sign monitoring, COVID-19 symptom assessments, twice-daily patient reporting, self-isolation education, centralized RPM RN care coordination and clinical assessments in response to alerts, and centralized COVID-19 care team physician and advanced practice provider support for nursing oversight. The high-intensity model offered a few unique features, including a blood-pressure cuff and monitor for blood pressure and heart rate vital assessments, up to four-times daily monitoring for immunosuppressed patients, and Spanish language support.

Clinical workflows were designed by the RPM team in collaboration with COVID-19 care teams for ambulatory patient enrollments and with Hospital Internal Medicine teams to support enrollment post discharge. The COVID-19 RPM program was implemented in the Mayo Clinic Midwest (Minnesota and MCHS) on March 26, 2020, Mayo Clinic Florida on April 13th, and Mayo Clinic Arizona on May 18th. The low-intensity monitoring program was discontinued on September 15, 2020, due to the overall favorable patient outcomes in this low-risk cohort and limited adoption across all sites.

For the COVID-19 RPM program, clinical support was provided 24 h per day, seven days a week, including weekends and holidays. The RPM RNs were centrally located in the Mayo Clinic Midwest. COVID-19 care teams comprising of physicians and advanced practice providers from primarily General Internal Medicine and Infectious Disease were located at each of the three main Mayo Clinic campuses and primarily responsible for RPM nursing oversight and all ambulatory COVID-19 patient management. RPM programmatic oversight was provided by a medical director, operations administrator and managers, and nursing administrator and manager within the Center for Connected Care.

Many new RPM RNs were recruited from the primary care and specialty practices to support the program’s rapid growth. Their orientation was streamlined to five days, and an online central repository was created to provide real-time access to updated resources and support for staff as care management guidelines changed quickly and frequently. A nursing workload measurement and reporting system^22^ helped nurse leaders develop a staffing model that incorporated the increased intensity of the COVID-19 RPM program that would incur as well as the indirect patient care time associated with nurses learning a new practice. Rapid fluctuations in the COVID-19 RPM patient census and workload per patient occurred throughout the pandemic and impacted care team workforce needs.

At all Mayo Clinic sites, screening and enrollment for the COVID-19 RPM program were completed by the centralized COVID-19 care teams with or without RPM nursing support. Upon confirmation of a positive SARS-CoV-2 test, patients were clinically assessed for initial hospital- or ambulatory-based management. For ambulatory patients, or those discharging from the hospital, RPM program intensity selection was determined by the COVID-19 care team assessment of risk factors for severe COVID-19 illness, as defined by the CDC and expert consensus (see Fig. 1). For those not meeting high-risk criteria, the low-intensity RPM care model was utilized; although at provider discretion, those patients who were asymptomatic or late in the disease course without risk factors were commonly not enrolled in either model of the RPM COVID-19 program. See Fig. 1 for the COVID-19 RPM program workflows and patient triage.

To foster broad access and inclusivity to the program, eligibility was expanded to support populations not previously served by the existing chronic condition program, including, but not limited, to those who were non-English speaking, without a Mayo Clinic primary care provider, with end-stage liver or kidney disease, or immunocompromised due to transplant, cancer, or other condition.

The RPM RNs utilize the technology to monitor patient dashboards and alerts for adverse health trends, as well as patient management guidelines and clinical assessment skills to deliver appropriate interventions aimed to provide supportive care in the home. When clinically indicated, care decisions were escalated to COVID-19 care team physicians and advanced practice providers. The COVID-19 care team physicians further maintained close contact with regional Mayo Clinic ED and Hospital Admissions and Transfers Coordinating Centers, such that ambulatory RPM patients requiring hospitalization could bypass the ED and be directly admitted to an inpatient COVID-19 unit. Additionally, they maintained close contact with the COVID-19 hospital internists and care managers to facilitate smooth care transitions for patients enrolling in the RPM program upon discharge.

The COVID-19 care team physicians established graduation criteria for the COVID-19 RPM program based on CDC guidance and expert consensus. Upon initial implementation, graduation, and subsequent release from isolation, patients required negative SARS-CoV-2 nasal swabs. As a greater understanding of COVID-19 infection, risk was gained and the CDC released new guidance^23–25^, program graduation criteria changed to reflect symptom-based criteria reflective of patient severity of illness. Patients were systematically assessed at the minimum isolation day, with monitoring extension for patients who did not fulfill clinical criteria for improvement. Early in the pandemic, patient retesting was coordinated by the COVID-19 care teams when indicated to ensure viral clearance, especially among healthcare professionals, as well as post-hospital and immunocompromised patients. This retesting practice was discontinued later in the pandemic due to evolving evidence against. RPM RNs were also able to evaluate and remove EHR COVID-19 infection status “flags” for patients thereby allowing easier and more timely return to routine medical care.

### COVID-19 RPM program analysis plan

This clinical research project and RPM program analysis were reviewed and approved by the Mayo Clinic Institutional Review Board (#18-009605). The COVID-19 RPM program evaluation was a cross-sectional retrospective medical record review focused on understanding the impact of an established RPM program rapidly adapted to meet the needs of patients during the pandemic and presented no greater than minimal risk for participants secondary to chart review. Authorization for retrospective record research was verified for any participant with medical records generated from care received in the state of Minnesota. Given these factors, the Mayo Clinic IRB approved a waiver of informed consent. Although both the high- and low-intensity RPM care models utilize different technology platforms to monitor patients, common assessments and measurements applied to and were used across both (Table 1).

The data for this study were abstracted from the electronic medical record. All Mayo Clinic sites utilize a single electronic medical record. Patients within the retrospective cohort included those who were enrolled into the Mayo Clinic COVID-19 RPM program between March 1^st^, 2020 and November 30^th^, 2020. The start date used for analysis was the RPM program enrollment date for patients enrolled from an outpatient setting or date of discharge for patients enrolled while hospitalized. Elixhauser comorbidity scores and risk factors were calculated for patients using ICD-10 diagnosis codes within one year prior to start date. Definitions for study endpoints related to the platform digital exchanges, including engagement, compliance, alerts, and escalations are provided in Table 4. Utilization and complication data were abstracted for a 30-day period beginning from the patient’s start date. Hospitalizations, ED visits, and ICU admissions were considered “all cause”, whereby they were included in the analysis irrespective of a direct attribution to the COVID-19. Complications were based on ICD-10 diagnosis codes previously identified as associated with COVID-19 infection^15^.

### Reporting summary

Further information on research design is available in the Nature Research Reporting Summary linked to this article.

## Supplementary information

Reporting Summary

## Data Availability

The data that support the findings of this study can be made available from the corresponding author (TCH) upon request of qualified researchers who meet the criteria for access to confidential data. The data are not publicly available due to inclusion of protected health information, ethical concerns about patient confidentiality, and disclosure limitations otherwise specified under the Privacy Rule (45 CFR Part 160 and Subparts A and E of Part 164).

## Code availability

The data for this study were abstracted from Epic (May 2020) via SAS (9.4), which was also used to perform the statistical analyses described in the Methods section. Data visualizations were produced using Tableau Server (2020.3.2), which subsequently used map data from Mapbox, OpenStreetMap, and their data sources (to learn more, visit https://www.mapbox.com/about/maps/ and http://www.openstreetmap.org/copyright). Any custom code can be made available from the corresponding author (TCH) upon reasonable request.
