# Supplementary material for: Implementation of a multisite, interdisciplinary remote patient monitoring program for ambulatory management of patients with COVID-19
Source: NPJ Digit Med. 2021 Aug 13;4:123. doi: 10.1038/s41746-021-00490-9 (PMC8363637; doi:10.1038/s41746-021-00490-9)
Supplement: Supplementary file 1 — Reporting Summary [file 41746_2021_490_MOESM1_ESM.pdf]

nature portfolio

Corresponding author(s): Tufia Haddad, M.D.
200 First Street S.W
Rochester, MN, 55905
Phone: 507-284-2511
Fax: 507-284-1803
Email: haddad.tufia@mayo.edu

Last updated by author(s): Jul 15, 2021

Reporting Summary

Nature Portfolio wishes to improve the reproducibility of the work that we publish. This form provides structure for consistency and transparency in reporting. For further information on Nature Portfolio policies, see our Editorial Policies and the Editorial Policy Checklist.

Statistics

For all statistical analyses, confirm that the following items are present in the figure legend, table legend, main text, or Methods section.

n/a	Confirmed	
☐	☒	The exact sample size (*n*) for each experimental group/condition, given as a discrete number and unit of measurement
☒	☐	A statement on whether measurements were taken from distinct samples or whether the same sample was measured repeatedly
☒	☐	The statistical test(s) used AND whether they are one- or two-sided<br>*Only common tests should be described solely by name; describe more complex techniques in the Methods section.*
☒	☐	A description of all covariates tested
☒	☐	A description of any assumptions or corrections, such as tests of normality and adjustment for multiple comparisons
☒	☐	A full description of the statistical parameters including central tendency (e.g. means) or other basic estimates (e.g. regression coefficient) AND variation (e.g. standard deviation) or associated estimates of uncertainty (e.g. confidence intervals)
☒	☐	For null hypothesis testing, the test statistic (e.g. *F*, *t*, *r*) with confidence intervals, effect sizes, degrees of freedom and *P* value noted<br>*Give P values as exact values whenever suitable.*
☒	☐	For Bayesian analysis, information on the choice of priors and Markov chain Monte Carlo settings
☒	☐	For hierarchical and complex designs, identification of the appropriate level for tests and full reporting of outcomes
☒	☐	Estimates of effect sizes (e.g. Cohen's *d*, Pearson's *r*), indicating how they were calculated

Our web collection on statistics for biologists contains articles on many of the points above.

Software and code

Policy information about availability of computer code

Data collection	The data for this study were abstracted from Epic (Epic Systems, version May 2020, Verona, WI), Mayo Clinic's electronic medical record, via SAS 9.4 software (SAS Institute, Cary, NC).
Data analysis	All analysis was performed using SAS 9.4 software (SAS Institute, Cary, NC).

For manuscripts utilizing custom algorithms or software that are central to the research but not yet described in published literature, software must be made available to editors and reviewers. We strongly encourage code deposition in a community repository (e.g. GitHub). See the Nature Portfolio guidelines for submitting code & software for further information.

Data

Policy information about availability of data

All manuscripts must include a data availability statement. This statement should provide the following information, where applicable:

- Accession codes, unique identifiers, or web links for publicly available datasets
- A description of any restrictions on data availability
- For clinical datasets or third party data, please ensure that the statement adheres to our policy

The data that support the findings of this study can be made available from the corresponding author (TCH) upon request of qualified researchers who meet the criteria for access to confidential data. The data are not publicly available due to inclusion of protected health information, ethical concerns about patient confidentiality, and disclosure limitations otherwise specified under the Privacy Rule (45 CFR Part 160 and Subparts A and E of Part 164).

Field-specific reporting

Please select the one below that is the best fit for your research. If you are not sure, read the appropriate sections before making your selection.

☒ Life sciences ☐ Behavioural & social sciences ☐ Ecological, evolutionary & environmental sciences

For a reference copy of the document with all sections, see nature.com/documents/nr-reporting-summary-flat.pdf

Life sciences study design

All studies must disclose on these points even when the disclosure is negative.

Sample size	8,548 patients were initially included within the data set for this program analysis, representing the census of patients who were enrolled into the RPM programs between March 26th - November 30th, 2020.
Data exclusions	7,074 were evaluable for this program analysis. 1,474 were excluded for the following reasons: 113 were excluded due to an unspecified program completion status, 382 patients were excluded because their program completion was outside of the evaluable period, 105 patients were excluded due to duplications within the dataset (a result of system artifacts), and 874 patients were excluded because they did not provide authorization for retrospective records research.
Replication	This study represents a retrospective descriptive analysis of a clinical program implementation rather than a prospective clinical trial.
Randomization	This study represents a retrospective descriptive analysis of a clinical program implementation rather than a prospective clinical trial.
Blinding	This study represents a retrospective descriptive analysis of a clinical program implementation rather than a prospective clinical trial.

Reporting for specific materials, systems and methods

We require information from authors about some types of materials, experimental systems and methods used in many studies. Here, indicate whether each material, system or method listed is relevant to your study. If you are not sure if a list item applies to your research, read the appropriate section before selecting a response.

Materials & experimental systems

n/a	Involved in the study	
☒	☐	Antibodies
☒	☐	Eukaryotic cell lines
☒	☐	Palaeontology and archaeology
☒	☐	Animals and other organisms
☐	☒	Human research participants
☐	☒	Clinical data
☒	☐	Dual use research of concern

Methods

n/a	Involved in the study	
☒	☐	ChIP-seq
☒	☐	Flow cytometry
☒	☐	MRI-based neuroimaging

Human research participants

Policy information about studies involving human research participants

Population characteristics	Relevant covariates captured and utilized within this analysis included age, sex, marital status, race/ethnicity, primary language, association with a primary care provider, COVID-19 testing location, month of COVID-19 test result, COVID-19 risk factors, Elixhauser score, and underlying clinical risk factors.
Recruitment	Patients were identified from among community-dwelling patients diagnosed as COVID-19 positive at one of the three main Mayo Clinic campuses or four Mayo Clinic Health System regions in the Midwest. 874 patients who enrolled in the program were excluded from this analysis because they did not provide authorization for retrospective records research (Disclosure of Health Records for External Research, Minnesota Statue §144.295, 2020).
Ethics oversight	This clinical research project and RPM program analysis were reviewed and approved by the Mayo Clinic Institutional Review Board (#18-009605). When applicable, authorization for retrospective records research was verified for any participant who has medical records generated from care received in the state of Minnesota. Additionally, this study presented no greater than minimal risk for participants secondary to chart review. Given these factors, the Mayo Clinic IRB approved a waiver of informed consent under 45 CFR 46.

Note that full information on the approval of the study protocol must also be provided in the manuscript.

Clinical data

Policy information about clinical studies

All manuscripts should comply with the ICMJE guidelines for publication of clinical research and a completed CONSORT checklist must be included with all submissions.

Clinical trial registration	This publication represents a retrospective analysis that doesn't otherwise qualify as a clinical trial and is not otherwise required to be registered in a public trials registry per ICMJE guidelines.
Study protocol	This publication represents a retrospective analysis that doesn't otherwise qualify as a clinical trial and is not otherwise required to be registered in a public trials registry per ICMJE guidelines. Study protocol "Remote Patient Monitoring and Association with Clinical Outcomes among Patients with Chronic, Sub-Acute, and Acute Conditions" was reviewed and approved by the Mayo Clinic Institutional Review Board (#18-009605).
Data collection	The data for this study were abstracted from the electronic medical record. Patients within the retrospective cohort included those with COVID-19 who were subsequently enrolled into one of Mayo Clinic's remote patient monitoring programs between March 1st, 2020 and November 30th, 2020. All Mayo Clinic sites utilize a single electronic medical record. The start date used for analysis was the RPM program enrollment date for patients enrolled from an outpatient setting or date of discharge for patients enrolled while hospitalized. Elixhauser comorbidity scores and risk factors were calculated for patients using ICD-10 diagnosis codes within one year prior to start date. Utilization and complications data were abstracted for a 30-day period beginning from the patient's the start date. Hospitalizations, ED visits, and ICU admissions were considered "all cause", whereby they were included in the analysis irrespective of whether there was a direct attribution to the patient's COVID-19 infection. Complications were based on ICD-10 diagnosis codes previously identified as associated with COVID-19 infection (O'Horo, J. C. et al. Outcomes of COVID-19 With the Mayo Clinic Model of Care and Research. Mayo Clinic Proceedings, doi:10.1016/j.mayocp.2020.12.006).
Outcomes	Given the descriptive nature of this publication, primary and secondary outcomes measures were focused on descriptive summaries of clinical, demographic, and program factors as well as summaries of commonly-measured clinical utilization. Many of these factors are generally reported relative to clinical program evaluation and/or were otherwise deemed associated with patients experiencing COVID-19 infection (O'Horo, J. C. et al. Outcomes of COVID-19 With the Mayo Clinic Model of Care and Research. Mayo Clinic Proceedings, doi:10.1016/j.mayocp.2020.12.006).
